# Hospital and Institutional News

**Published:** 1918-08-03

**Authors:** 


### HOSPITAL AND INSTITUTIONAL NEWS.

### THE CENSUS OF MEDICAL STUDENTS.

The General Medical Council, which was requested by the Minister of National Service to make a periodical census of the students actually attending the medical schools of the United Kingdom, has provided lists for January 1917, October 1917, and May 1918. In January 1917, 6,682 medical students were in training. In October last year this number had risen to 7,048, and by May last the total reached 7,630. Of these two-thirds are men and one-third women. Latterly, of course, the proportion of women students has increased. The number of first-year men in May was nearly 1,400, and if they duly qualify in 1922 the nominal number of accessions to the Register before the war will have been supplied. It is therefore hoped that the supply of doctors for the future has been secured by the recent measures, even though medical studies at present have necessarily to be combined with military duty.

### HASLAR THE GREAT.

Like many remarkable institutions, Haslar Hospital is little known to outsiders. Few are aware that it is a colossal institution, with nearly sixty acres of land, and 1,346 beds, of which 152 are reserved for officers. Yet it is doubtful if any other hospital performs such varied work, though whether this centralisation is wholly desirable is perhaps open to question. At all events, tuberculosis, skin diseases, zymotic diseases, and mental diseases (as Dr. Mercier would not call them) are treated in this one place. Besides there is the vast store which supplies the fleets whose base is at Portsmouth. The hospital museum is of very varied interest, for its contents have been collected in every part of the world to which naval medical officers penetrate. Not content with its own exchange, the institution has its own post office and its own theatre. It is, in fact, a miniature hospital city.

### THE BRITISH SCIENCE GUILD.

From August 12 to September 7 there will be held at King's College, London, a British Scientific Products Exhibition, which no intelligent man should miss. With the approval of the Board of Trade and the assent of the Ministry of Munitions, the exhibition will display a collection of products and appliances of scientific and industrial importance which, before the war, were obtained chiefly from hostile countries, but which are now produced in the United Kingdom. The principal object in view is twofold. First to apprise the public how the combination of science and industry has enabled manufacturers to catch up the lead which the Germans possessed. Secondly, how our newly won independence in this field can be established and maintained in the future. The exhibits naturally fall into three groups: Those which even before the war we were able to make and export to foreign markets; those which, previously imported, are now being manufactured here; and those in the manufacture of which considerable advance has been made during the war. It is believed that the display will stimulate scientific and industrial research, and help to improve our manufactures. The chemical products will include synthetic dyes, non-ferrous metals, and pharmaceutical preparations. The physical appliances will illustrate pyrometers, laboratory furnaces, and high-temperature thermometers, optical and cinematograph apparatus, vacuum gauges, while surgical, bacteriological, and pathological appliances will have a section to themselves. Paper, including photographic and filter papers, will be exhibited, and so will the success attained in exploiting British natural products. Similar exhibitions have been held in France and in America, and the British Science Guild is to be congratulated on its initiative in the matter.

### A MODEL HOSPITAL FAIR.

We have always held that every undertaking which is carried out on behalf of a hospital, even a concert or matinée or fête, should be distinguished from the common run of similar enterprises by the possession of an unmistakable hospital air. It is far from easy to translate this desire into action, but we note that it was accomplished in some degree last week at the Musical Fair which was held at, and on behalf of, the Royal Free Hospital. For example, the stalls were partly furnished by the small neighbouring shops in Gray's Inn Road, former women patients brought their families to make purchases at the stalls presided over by the women doctors and the hospital sisters, and officer patients from the military wards received the admission moneys at the gate. Doctors and students in fancy costume acted as saleswomen in "Garden Square," as the place deserved to be called from the flowers, fruit, ducks, and chickens which filled it. The model aeroplanes and transport wagons which were on sale had been made by the inventive patients of the 1st London General Hospital, Wandsworth. One of the stalls was that of the Appeal Committee, but it did not sell "appeals" among its books, though with time for research a list of the best and shortest would make an entertaining volume. Mr. J. H. Dowd, with whose sketches our readers are familiar, made lightning likenesses of people, and portraits of the staff were sold by Lieutenant L. J. Smith. Ten-minute lectures were given. Microbes were on show, and the hospital atmosphere permeated everything. The stalls were finally cleared by the munition girls who trooped in during the evening. The extension fund deserves to benefit accordingly.

### A MEDICAL OFFICER AND WARRIOR ORPHANS.

Durham County Council has appointed Dr. Elspeth Fowler as whole-time lady medical officer in connection with maternity and child welfare work, and she expects to take up her duties on September 1. According to the quarterly report of the Durham County Superintendent Health Visitor, the health visitors have been asked to undertake the home supervision of motherless children of deceased Service men, and she observes that the matter is one of considerable importance as the pension—10s. weekly for the first child and 9s. 2d. for the other children—though no more than is necessary, yet bulks largely in the family budget of some of the families having the care of the children. The liberal pension is paid to ensure that the children are properly reared, and anything in the nature of "child-farming" must be repressed.

### PROPOSED FEDERATION OF WAR CHARITIES.

PEOPLE in the United States are very sensibly wishing to learn by the mistakes of this country in the matter of the organisation of war charities, and to prevent the waste that is bound to arise out of overlapping and too indiscreet activity. One suggestion is made by the American Association for Organising Charity which has caused some interest and no little debate. It is, briefly, to start a War-Chest Campaign—a scheme for collecting in one campaign all of the funds the many war organisations are expected to need in the course of a year. The scheme is not meeting with much enthusiasm, probably, we should think, because it is too much like taxation and too little like charity, and the interest that is given to the individual donor through knowing within a little what his money is going to be spent upon is entirely lost. As some super-organisers go a little further and wish to include ordinary social efforts in the omnibus charity, we are not surprised that the associate director of the New York C.O.S. considers that, as under this plan social work would be financed, not on its merits, but through enthusiasm engendered for war work of all kinds, social work would be cut off for the period of the war from that vital contact with its supporters which now comes through their gifts to the organisations in which they are interested.

### FOOD CONTROL AND ASYLUM DEATH-RATES.

AN important matter is referred to in a report which the Board of Control Commissioners have presented to the Birmingham Corporation with regard to the City Mental Hospital. The Commissioners refer to the high death-rate and say: "It is extremely undesirable that public confidence in the institutional treatment of the insane, which took so long to establish, should be shaken by the knowledge that the death-rate of even one asylum should approximate to 30 per cent. of the daily population without strenuous effort being made to reduce it." They proceed to suggest that food restrictions are not unconnected with the death-rate, observing: "The more healthy condition of the asylum now, as compared with its state up till recently (coincident as it is with better facility for obtaining food), seems to point to dietetic restriction as a potent causative influence in the production of a high sickness and death rate. This is, therefore, a factor that needs careful study and investigation by the committee during the period when circumstances are favourable, in order that preparation may be made for next winter, when conditions may again prove adverse. If certain staple articles of diet are likely to be unobtainable every effort must be made to ensure a supply of substitutes, as has been done successfully in some other institutions." A death-rate of 30 per cent. in an asylum calls for prompt investigation on the part of the Board of Control and the Birmingham Corporation.

### LUNATICS AS ATTENDANTS.

REPORTS of Board of Control Commissioners on the Birmingham City Asylum mention that the staff at present (June 1918) consists of eleven male and ten female charge officers, twenty-three ordinary attendants, twenty-nine nurses, and five male and six female night attendants and nurses. No nurses are employed on the male side, but the Visiting Commissioners understand that arrangements are being made to instal them as soon as necessity indicates their appointment. In the meantime, and for many months past, Dr. Roscrow, the superintendent, has supplemented his male staff, satisfactorily, by the employment of patients to the number of six as assistant attendants. The Commissioners, who saw these men, say they consider that the experiment may be regarded as successful.

### THE LONGEVITY OF DOCTORS.

THE death of two surgeons in the North of England last week shows that longevity can attach to the medical profession. Mr. George Blacker Morgan, who died on July 25 at Sunderland, aged eighty-four, for over sixty years had been a surgeon at the Royal Infirmary. Mr. Thomas Walker, who died on the same day at Chapelthorpe Hall, near Wakefield, aged ninety-two, also had a life-long association with institutions in that part of the West Riding, dating from his appointment as house surgeon at the Wakefield Dispensary in 1852. He held this post for three years, being in 1856 appointed honorary surgeon at the Clayton Hospital, and serving in that capacity until his retirement from practice in 1892, when he was made consulting surgeon. One of his sons is Mr. Secker Walker, an ophthalmologist at Leeds.

### THE SALARY OF MEDICAL SUPERINTENDENTS.

A TRIBUTE to the work done by Colonel E. Goodall for the Cardiff Mental Hospital during the past ten years was paid by the chairman of the committee, Alderman Morgan Thomas, and others of the members, when the question of the superintendent's salary was considered on July 25. After some discussion it was unanimously decided to raise Colonel Goodall's salary immediately from £800 to £900 a year, with another increment of £50 next January, and a further £50 in the January following.

### SAFEGUARDING POSTS FOR ABSENT DOCTORS.

THE Local Government Board has declined to sanction the permanent appointment of a district medical officer to the Wigan Board of Guardians. In communicating this decision, Mr. Stephen Walsh, Parliamentary Secretary of the Board, declared its policy as follows: "The general policy is that such appointments should be sanctioned for the period of the war only. The country is under an obligation not in any way to diminish the opportunity of medical men who have given their services to the nation to secure employment upon their return to civil life." Unfortunately, private practice cannot be preserved in the same way.

### COMPLAINTS AGAINST A WELSH HOSPITAL JUSTIFIED.

The sub-committee of the Glamorgan Insurance Committee appointed to investigate complaints made by a Caerphilly patient, Miss Edith May Powell, against the Glan Ely Hospital, has found the complaints justified. Since the patient was too ill to attend a meeting at the hospital, a deputation visited her home to take her evidence and that of her parents. Dr. Brownlee and Dr. Hiley, of the Welsh National Memorial Association, and Sister Finlayson, of the Glan Ely Hospital, were also present. At a meeting of the Glamorgan Insurance Committee held on July 25 the sub-committee presented its report, which contained the following: "After carefully considering the evidence, we are satisfied there is ground for the complaints made, more especially the one having reference to lack of cleanliness. We took exception to the type-written declaration which has to be signed by patients leaving against advice, and it was understood that a new form would be utilised in future." Alderman D. H. Williams, a member of the sub-committee, said the form contained a sentence to the effect that the patient "had no cause for complaint." Mr. W. P. Nicholas, chairman of the Insurance Committee, strongly condemned the form referred to, remarking that it required a person of strong moral courage to refuse to sign a document because he or she was dissatisfied with the dietary or the cleanliness of the hospital. It was decided to forward a copy of the sub-committee's report to the Welsh National Memorial Association, and to request the abolition of the offending form.

### FREE FOOD FOR POOR MOTHERS.

Southwark Borough Council has sanctioned a scheme submitted by Dr. G. Millson, L.R.C.P., M.R.C.S., Medical Officer of Health, for providing food and milk for mothers and infants in cases of necessity. The assistance is to be confined to families where the total income is under 30s. a week with three children, and 35s. with more than three children. Dr. Millson estimates the number of children born yearly in the borough at 3,500, and that 2 per cent. of the mothers, that is seventy, might be taken as the number in a state of poverty. These, he suggests, should be provided with dinners daily for three months, costing 6d. each, which would involve an annual expenditure of £164. The milk, he estimates, would cost a further £136, or £300 altogether, and, as half the amount will be refunded by the Local Government Board, the borough would only be involved in an annual expenditure of about £150. The whole question of supplying milk and food must be approached with great care, and Dr. Millson has assured the Public Health Committee that no order will be issued except upon his personal signature or that of some accredited representative of the Council. Primarily the object of the scheme is the preservation of infant life, and, as this is the first attempt of a metropolitan Borough Council south of the Thames to grapple with the question, it is to be hoped that Dr. Millson's scheme will prove a success.

### THE WORK OF SANATORIUM PATIENTS.

At a recent meeting of the Bristol Insurance Committee, Mr. J. L. Brown gave an account of Winsley Sanatorium. Probably a considerable number of beds will soon be added. Under the Farm and Gardens Committee the employment of patients would form a link with the after-care treatment for those whose sanatorium treatment had been concluded. The work already done by Dr. Rees, the medical superintendent, was praised by Mr. Brown. He spoke of the objections raised by the working classes to work at a sanatorium, but the work was good for the patients. At Winsley they were divided into three classes. For one, absolute rest was prescribed; the second class took exercise by walking, and from that the patients were graded for various kinds of suitable work. To show the variety of work performed, Mr. Brown said that a piece of roadway about 300 yards long had been made: the old road was dug to a depth of eighteen inches, the rubbish wheeled away, when a solid foundation of stone, cracked by patients, was laid down. An orchard of 140 trees had been planted. A piece of rough ground, about 40 yards square, had been levelled and turfed. Part of the excavation was through rock, which was broken with sledge-hammers and wedges. The men on heavy work built a containing wall at the bottom of a stone tip. It was a formidable piece of work, and involved the handling of many tons of stone. In the gardens, after the production of sufficient vegetables for the sanatorium, there was a surplus for disposal, and in 1917 a net profit of £100 was made.

### THE UNINSURED AND SANATORIUM TREATMENT.

The difficulties which confront any administrator anxious to put his sanatorium to the most fruitful use are plain from recent experiences at Kelling Sanatorium, Norfolk. Dr. W. J. Fanning, the medical superintendent, and the committee, for example, often receive applications for admission from Insurance Committees, although the sanatorium was founded and maintained, as the report records, for those who are lucky enough to come within the machinery of the hated Insurance Act. In an endeavour not to admit "hopelessly unsuitable" cases, the committee was confronted with several applications from sailors and soldiers the condition of whom was unfortunately in some cases beyond effective aid. The men are admitted, however, until it was shown that they were gaining no benefit, when they were discharged. The need of these unfortunate men, coupled with the fact that an ordinary sanatorium for improvable cases is not intended for advanced cases, leads us to hope that before long special institutions for men of the Forces who are in similar case may be founded, and that this may prove a step towards the long-overdue establishment of institutions for advanced cases only.

### HIGHER SALARIES AT WORCESTER INFIRMARY.

The committee of Worcester General Infirmary has approved the following increases of salary to members of the hospital staff:- the acting resident medical officer, Mr. Allport, is to have £250, as from April last, instead of £160; a war bonus of £3 per month is to be given to Mr. E. J. Holland, the secretary; a war bonus of £10 per annum is to be given to Sister Watson, the acting matron; and a war bonus of £3 a year is to be added to the existing bonuses granted to the sisters, nurses, and probationers. Those granted to Mr. Holland and Sister Watson were unsolicited, but were given by the committee in view of the increases granted to the nursing staff. To secure additional subscriptions and donations Miss Ashby has been appointed canvasser, with instructions not to trespass upon the field occupied by the Saturday Fund and the penny monthly collection scheme. Miss Ashby has had previous experience of canvassing on behalf of Kidderminster Infirmary.

### LOTTERIES FOR CHARITY.

The Bill presented by Lord Lansdowne in the House of Lords to legalise in certain instances lotteries on behalf of war charities is restricted to the duration of the war. The appeal of betting, or some game of chance, is human and natural, like the taste for wine, and as such should be respected and controlled rather than repressed. Where charities are concerned, however, the vulgar prejudice against lotteries has to be considered, and in the case of the voluntary hospitals we believe that they can easily afford to be independent of this means of raising money, though whether the Government might not advantageously adopt public lotteries is another matter.

### TWO METHODS OF APPOINTMENT.

In these days, when hospital management is becoming a recognised profession, when its status, remuneration, and preliminary training are often discussed, a recent story told by Surgeon-General Sir L. Gubbins at the Devonshire Hospital, Buxton, will be listened to with mixed feelings. The speaker related that many years ago a friend of his called upon the Duke of Cambridge and asked to be appointed to a musical post. In answer to inquiries he confessed that he did not know a note of music. He was appointed, however, and, Sir L. Gubbins added, proved a conspicuous success. This success, the speaker suggested, was due to the fact that the man was able to take a detached view, and to look upon the institution to which he was appointed with an unbiased mind. We infer that the post was mainly administrative, and that the profession of music at that time, to say the least, was not more highly organised than that of institutional management to-day. The democratic notion forbids this kind of experiment, which, when successful, is the glory of patronage. The lesson of the story seems to be that "to go through the mill" can be as foolish a panacea for ineptitude as to insist on a University education.

### ASYLUMS BOARD NURSES AT BUCKINGHAM PALACE.

The Ministry of Labour invited the Metropolitan Asylums Board to take part in the Women's Procession which presented the Address of Homage to the King and Queen at Buckingham Palace. The arrangements were made by the Institution Sub-Committee, and a contingent of nurses, including eight matrons and assistant-matrons, ambulance drivers, and the domestic staff, represented the Board. Miss F. M. Ambler-Jones, of the South-Eastern Hospital, the senior matron of the party, had the honour of being presented to their Majesties.

### THE DICKENS HOME FOR THE BLIND.

Under the joint patronage of the Dickens Fellowship and St. Dunstan's, a Charles Dickens Home for blind sailors and soldiers is being established at Bannow, St. Leonards. The house and garden have been acquired, and the object of the Dickens Fellowship is to equip and to endow it. The members of the Fellowship point out that the characters of whom Dickens wrote have their modern counterpart in the Services to-day; and the memory of Dickens, who actually led one hospital appeal for another type of incurable case, and was interested in many, would be honoured after his own heart in seeing the new home carried through to complete success.

### THIS WEEK'S DRUG MARKET.

The chief event of the week is a drop of something like 30 per cent. in the price of potassium bromide; a reduction in price had been expected, and readers of these reports had been prepared for it and had no doubt refrained from buying more than sufficient to carry them over from week to week. The reason of the reduction is the importation of considerable quantities from the United States, which have been distributed to the wholesale houses, etc., without passing through the open market. Ammonium bromide and sodium bromide are unchanged in price; the ammonia salt is now very much dearer than potassium bromide, and even the sodium salt costs almost as much as the potassium salt. We may therefore expect to see a falling off in the demand for ammonium and sodium bromides, which will relieve the pressure until these salts are in better supply and possibly cheaper. It seems likely that sugar of milk will be brought under some form of Government control. Iodides are in good demand. A fair business continues to be done in imported saccharin. The good demand for iron and quinine citrate cannot be met by the makers. Quinine still tends upwards in price, but the demand is quieter. Lithia salts are scarce. The scarcity of glycerophosphates has become very acute, and, in view of the absence of glycerin, there is no likelihood of any relief. Ergot of rye is rather lower in price, being more freely offered. American peppermint oil is getting still scarcer, and the price is advancing.

